# A Rare Manifestation of Unknown Hyperparathyroidism as a Perforated Peptic Ulcer

**DOI:** 10.7759/cureus.37635

**Published:** 2023-04-16

**Authors:** Gideon Mlawa, Zahid Khan, Sabeen Azhar, Furhana Hussein, Bashir Mahamud, Abdulrahman Nugod

**Affiliations:** 1 Internal Medicine and Diabetes and Endocrinology, Barking, Havering and Redbridge University Hospitals National Health Services (NHS) Trust, London, GBR; 2 Acute Medicine, Mid and South Essex National Health Services (NHS) Foundation Trust, Southend on Sea, GBR; 3 Cardiology, Bart’s Heart Center, London, GBR; 4 Cardiology and General Medicine, Barking, Havering and Redbridge University Hospitals National Health Services (NHS) Trust, London, GBR; 5 Cardiology, Royal Free Hospital, London, GBR; 6 Acute Medicine, Queen's Hospital, London, GBR; 7 Critical Care Medicine, Queen's Hospital, Romford, GBR

**Keywords:** acute abdomen in covid-19, urgent parathyroidectomy, mibi scan, air under the diaphram on chest x-ray, peptic ulcer perforation (pulp), hyperparathyroidism treatment, adult primary hyperparathyroidism, severe hypercalcemia, acute hypercalcemia

## Abstract

Hypercalcemia is a common electrolyte abnormality with different causes. Hypercalcemia is most often associated with malignancy and primary hyperparathyroidism and malignancy together account for most cases. Primary hyperparathyroidism manifests as hypercalcemia owing to the overproduction of parathyroid hormone. In most cases, primary hyperparathyroidism manifests due to a solitary parathyroid adenoma. Based on calcium levels, hypercalcemia can be classified as mild, moderate, and severe. Hypercalcemia typically presents with non-specific clinical features. Here, we present the case of a 38-year-old male patient who presented to the emergency department (ED) with acute abdominal pain and a tender abdomen with absent bowel sounds. He had chest radiography and blood tests initially. Chest radiography showed left-sided pneumoperitoneum, and the patient was suspected to have a perforated peptic ulcer due to hypercalcemia secondary to a parathyroid adenoma during the second wave of the coronavirus disease 2019 (COVID-19) pandemic. The findings were confirmed by a computerized tomography scan of the abdomen, and the patient was treated with intravenous fluids for hypercalcemia and was managed conservatively for a sealed perforated peptic ulcer following discussion in the multi-disciplinary team meeting (MDT). The COVID-19 pandemic led to a long waiting list and delays in the timely management of patients requiring elective surgical intervention, such as parathyroidectomy. The patient made a complete recovery and had parathyroidectomy of the inferior right lobe two months later.

## Introduction

Calcium is an essential cation, and almost one-half of the total serum calcium is bound to plasma proteins, predominantly albumin [1]. The remaining one-half circulates as ionized calcium, and it is this half of calcium that is physiologically active taking part in several processes including but not limited to cellular transport, muscle contraction, membrane function, clotting, nerve impulse conduction, and regulation of heartbeat [1,2]. Calcium concentration in the body is tightly regulated within a range of 2.2-2.5 mmol/L (8.5 to 10.5 mg/dL) by the parathyroid gland, which secretes parathyroid hormone (PTH) in response to low levels of circulating calcium. Conversely, an increase in calcium levels inhibits the release of PTH through a negative feedback loop. Hypercalcemia is clinically diagnosed when there is an increase in serum calcium levels above the upper limit of >2.5 mmol/L (10.5 mg/dL) on two separate occasions [3-6].

Hypercalcemia is considered mild when the total serum calcium level measures from 2.63 to 3 mmol/l (10.5 and 12 mg/dL), whereas levels greater than 3.5 mmol/l (14 mg/dL) are considered high and lead to the so-called hypercalcemic crisis, which is a life-threatening condition [6]. The etiological factors of hypercalcemia can be grouped into two main categories: PTH-dependent and PTH-independent processes. The PTH-dependent factors include primary hyperparathyroidism (PHPTh) due to parathyroid adenoma or hyperplasia, familial hypocalciuric hypercalcemia (FHH), tertiary hyperparathyroidism, and in some rare cases, malignancy. PTH-independent mechanisms include malignancy-associated hypercalcemia, granulomatous disorders (predominantly sarcoidosis), thyrotoxicosis, poorly monitored drug therapies (thiazide diuretics, calcitriol, and lithium therapy can lead to lithium-induced PHPT), excessive ingestion of calcium carbonate ("milk-alkali syndrome"), vitamin D toxicity, and immobilization [7-10].

The clinical features of hypercalcemia are non-specific and can manifest as fatigue, abdominal pain, nausea and vomiting, bone pain, polyuria, and, in severe cases, lead to coma. Hypercalcemia can also result in cardiac arrhythmias, acute kidney injury, and nephrogenic diabetes insipidus. Peptic ulcer disease (PUD) is a known complication of hyperparathyroidism, but peptic ulcer perforation (PUP) is rare and has only been reported in a few cases [10-12]. Perforated peptic ulcer (PPU) is a serious complication of PUD, and PUP often leads to the acute abdomen, a serious medical emergency that is associated with high morbidity and mortality [13]. Herein, we report the case of a patient who presented with a sealed PPU and signs of acute abdomen due to hypercalcemia resulting from a parathyroid adenoma.

## Case presentation

**Figure 1:**
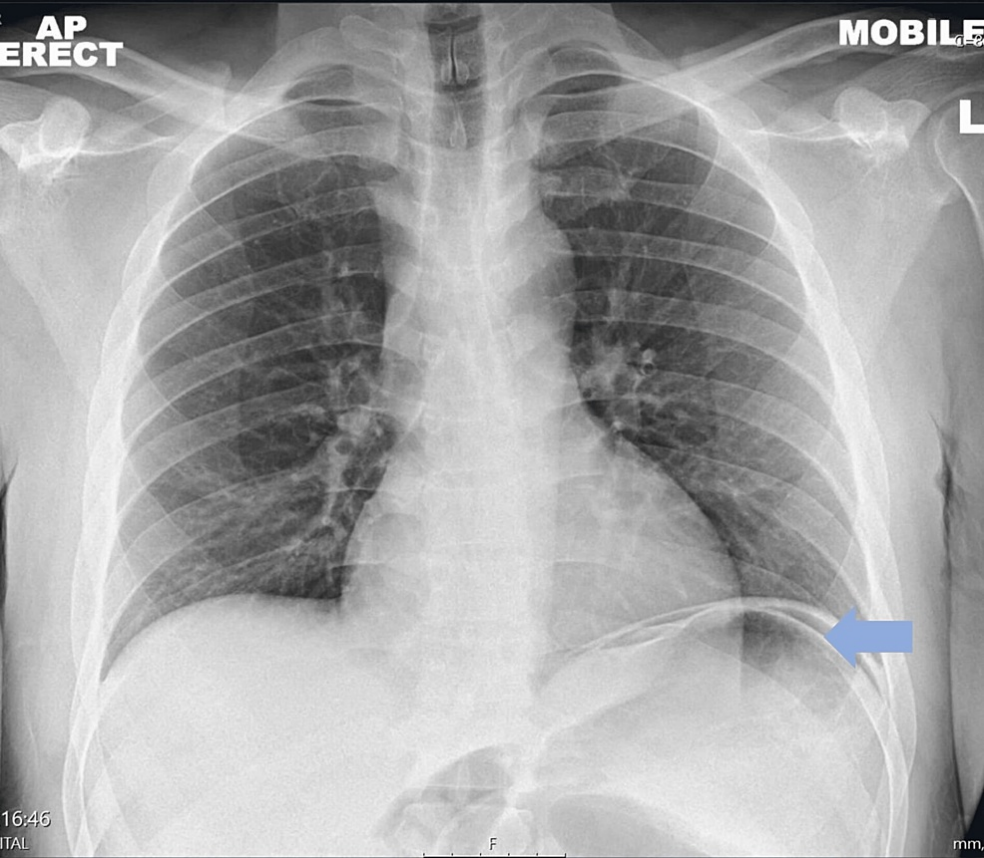
Chest radiography demonstrating air under the diaphragm highlighted by the colored arrow.

**Figure 2:**
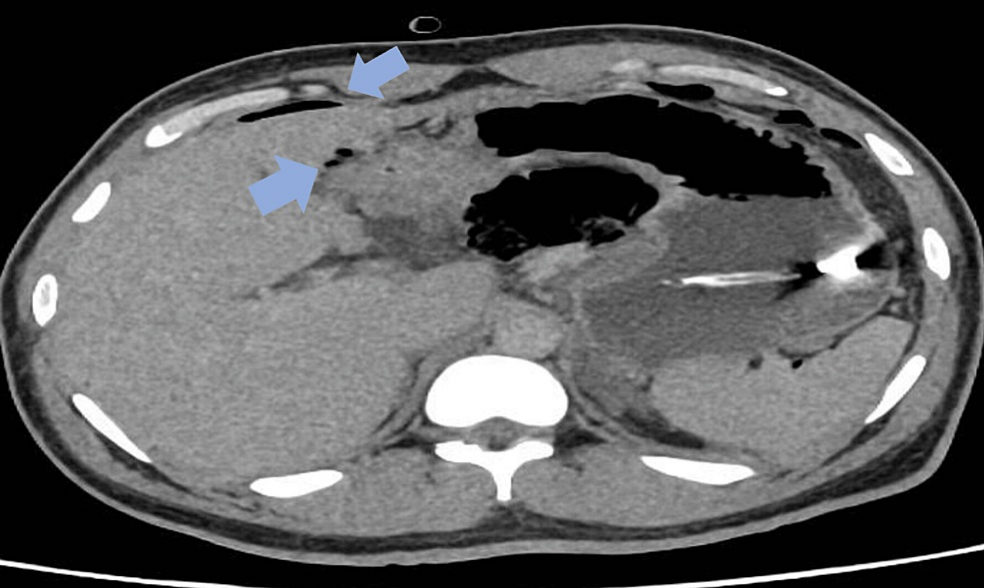
Computerized tomography scan of the abdomen and pelvis demonstrating mild pneumoperitoneum with localized air loculi adjacent to the pylorus and the first part of the duodenum (colored arrow).

A 38-year-old gentleman was admitted to the hospital with a three-day history of severe abdominal pain accompanied by nausea and vomiting. Clinical examination demonstrated abdominal distension, tenderness mainly epigastric, guarding, and the absence of bowel sounds. He was hemodynamically stable and was only slightly tachycardic with a heart rate of 105 bpm. Laboratory results showed high calcium and PTH levels raising the suspicion of primary hyperparathyroidism (Table 1). The patient had an urgent abdominal x-ray and chest x-ray that showed free air under the diaphragm, which led to clinical suspicion of gastric ulcer perforation (Figure 1). Following this, an urgent CT abdomen was performed, which demonstrated pneumoperitoneum with localized air loculi adjacent to the pylorus and the first part of the duodenum consistent with a perforated peptic ulcer (Figure 2). He was hemodynamically stable, and the case was discussed with the surgical on-call team, who believed that this was a sealed perforated peptic ulcer and wanted a multi-disciplinary team meeting (MDT) discussion. The patient was discussed in MDT the next day, and the consensus about the findings was it to be a sealed perforated peptic ulcer, and a conservative approach was advised in this case. The patient had a nasogastric tube (NGT) inserted and was given intravenous (IV) antibiotics, analgesia, and fluids with a regular clinical review.

**Table 1:** Lab values for the patient on admission.

Test	Result	Reference range
Hemoglobin	132	135-170 g/L
White cell count	6.2	3.5-11 × 10^9^/L
Platelet	355	140-400 × 10^9^/L
Neutrophils	6.3	1.7-7.5 × 10^9^/L
C-reactive protein	108	0-5 mg/L
Amylase	117	30-110 U/L
Parathyroid hormone	18.9	1.6-6.9 pmol/L
Na	136	135-145 mmol/L
Potassium (K)	4.3	3.5-5.1 mmol/L
Urea	2.5	2.9-8.2 mmol/L
Creatinine	75	66-112 umol/L
Calcium	2.94	2.2-2.6 mmol/L
Phosphate	0.53	0.8-1.5 mmol/L
Magnesium	0.78	0.85-1.10 mmol/L
Vitamin D	18	>50 nmol/L

**Figure 3:**
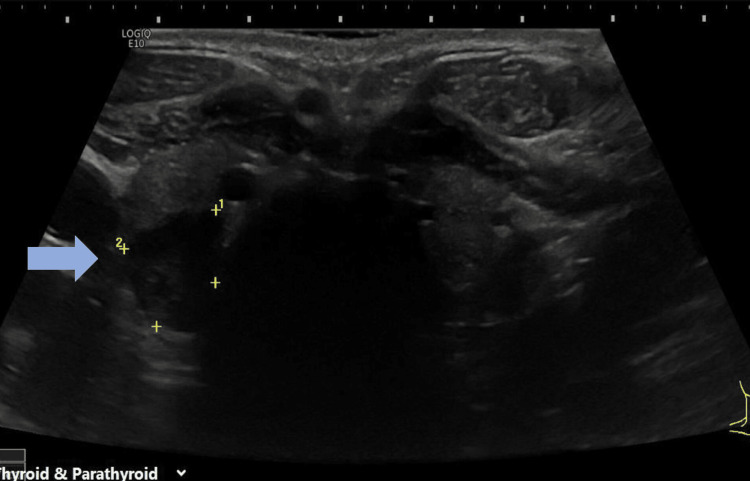
Ultrasound shows a parathyroid adenoma (17.5 mm × 10.5 mm × 8.5 mm) that lay adjacent to the lower pole of the right thyroid lobe.

**Figure 4:**
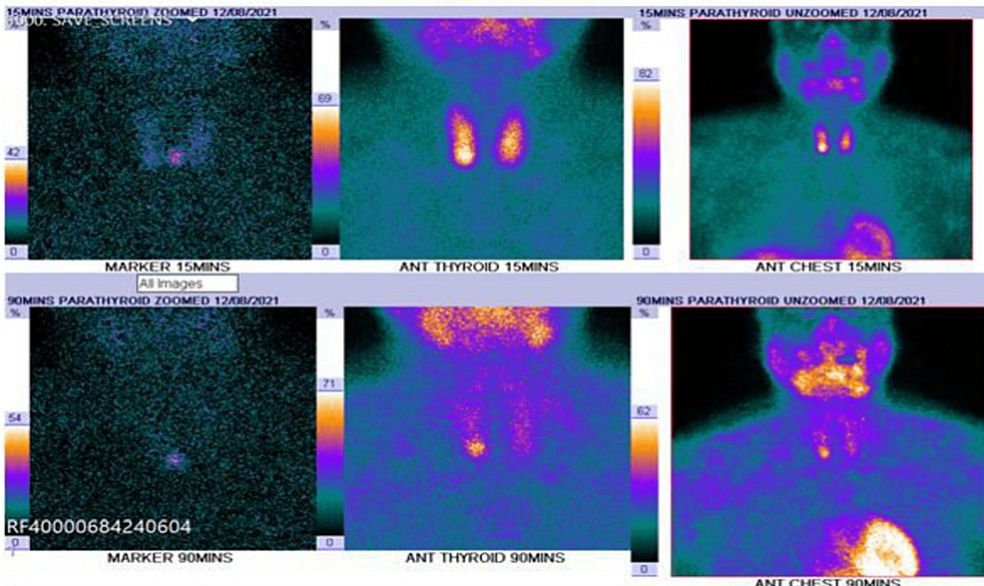
Sestamibi parathyroid scan showing uptake of radioactive material in the parathyroid gland.

Initial blood investigations also showed high C-reactive protein (CRP), elevated calcium, and high PTH levels but normal renal functions (Table 1). Ultrasound of the neck showed the presence of a parathyroid adenoma (17.5 mm × 10.5 mm × 8.5mm) that lay adjacent to the lower pole of the right thyroid lobe (Figure 3). Following this, the patient had ^99m^Tc-sestamibi scans with single photon emission computed tomography (SPECT) of the parathyroid gland, which demonstrated visible uptake within 15 min after administration of the nuclear agent uptake in the thyroid gland, and 90 min later, the uptake was concentrated mostly in the right inferior parathyroid gland indicating the presence of the parathyroid adenoma (Figure 4).

**Figure 5:**
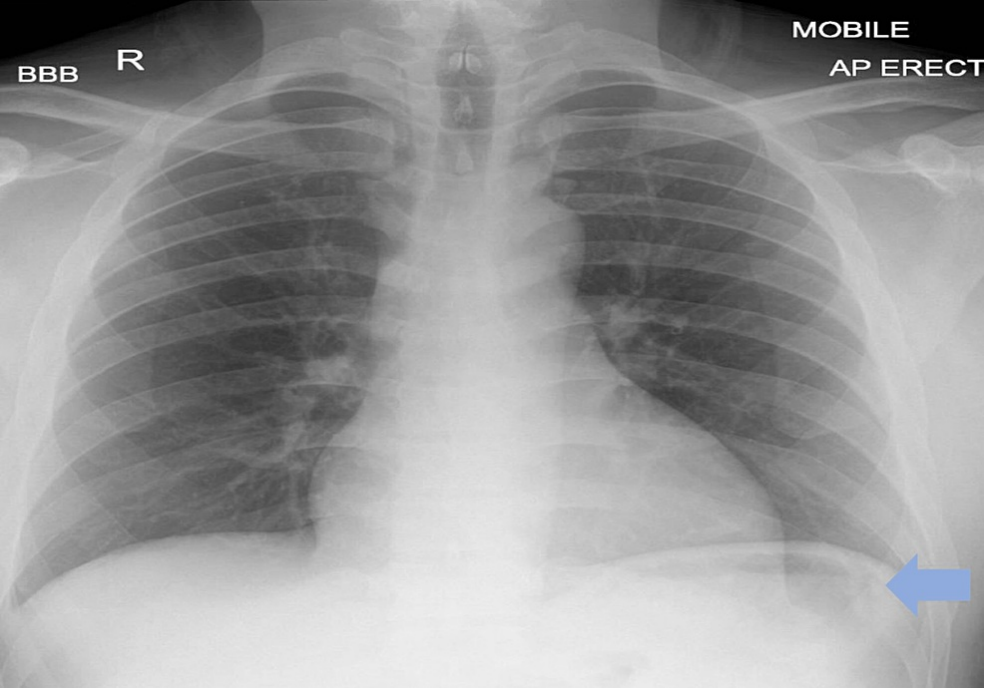
Repeat chest radiography shows a very small residual pneumoperitoneum.

While in the hospital, the patient’s calcium improved slightly with intravenous (IV) fluids (Table 2). His peptic ulcer perforation was treated conservatively, and a repeat chest x-ray showed significant improvement in the size of the pneumoperitoneum (Figure 5). He was discharged home after 10 days of admission following a discussion in the endocrine and surgical MDT for surgical resection of parathyroid adenoma as an outpatient. Following a multi-disciplinary team review, surgical parathyroidectomy of the inferior right parathyroid gland was advised. 

**Table 2:** Shows the elevated calcium levels of the patient during his stay at the hospital.

Adjusted calcium levels		
Date	Result	Reference range
04/05/2021	2.94	2.2-2.6 mmol/L
05/05/2021	2.87	2.2-2.6 mmol/L
08/05/2021	2.74	2.2-2.6 mmol/L
09/05/2021	2.79	2.2-2.6 mmol/L
10/05/2021	2.79	2.2-2.6 mmol/L
11/05/2021	2.85	2.2-2.6 mmol/L

Two months later, the patient underwent elective parathyroidectomy of the inferior right lobe (measuring 20 × 12 × 11 mm and weighing 1.01 g). He became clinically stable post-operatively, with normalization of calcium and was discharged home with an outpatient upper gastrointestinal endoscopy in six weeks by the gastroenterology team and a review by the endocrine team. This case highlights the risks associated with untreated hyperparathyroidism, and patients can present with acute abdomen due to hypercalcemia secondary to hyperparathyroidism.

## Discussion

Primary hyperparathyroidism is one of the most common endocrine disorders, with an incidence of 0.4 to 82 cases per 100,000 [14]. The diagnosis of PHPTh is usually made incidentally with an initial finding of hypercalcemia on routine laboratory studies, which leads to further investigation. Commonly, primary hyperparathyroidism is due to a solitary parathyroid adenoma [15]. Most cases of hyperparathyroidism are asymptomatic, but several patients manifest with hypercalcemia, which is characterized clinically by a pentad of symptoms, including stones, bones, groans, and psychic moans [15].

Notably, hypercalcemia can lead to alteration to the function of excitable membranes leading to skeletal muscle and gastrointestinal smooth muscle fatigue. A rare but clinically important manifestation of hypercalcemia is the development of a PUD. It is reported that up to 12% of patients with primary hyperparathyroidism have peptic ulcer-related symptoms [12,16,17]. The mechanism of how hypercalcemia leads to PUD is not well established, but several studies back the notion that excess calcium activates the stomach calcium-sensing receptor located on the basolateral membrane of gastric parietal cells, which in turn increases gastric acid secretion [12,18,19]. PPU is a serious complication of PUD, and patients with PPU often present with an acute abdomen, which has a high morbidity and mortality rate.

In the case presented here, the patient had undiagnosed hypercalcemia secondary to hyperparathyroidism presenting with signs of a perforated peptic ulcer. High clinical suspicion of hyperparathyroidism developed because of persistently elevated serum calcium levels and high serum PTH, which were confirmed by imaging. The key learning point from this case report is the fact that hyperparathyroidism can also present as a surgical emergency in certain cases, and clinicians need to be aware of this. He had to wait two months for surgery unfortunately during the second wave of the COVID-19 pandemic to undergo parathyroidectomy. 

Previous case reports have similar findings, wherein patients presented with acute abdomen features secondary to undiagnosed hyperparathyroidism [12,16]. A 48-year-old patient presented to the hospital with sudden onset acute mid-epigastric abdominal pain accompanied by nausea and symptoms of dyspepsia and nausea for 15 days [12]. This patient had rebound tenderness, guarding, and absence of bowel sounds on the clinical examination, and a chest radiograph showed pneumoperitoneum just like our patient. The patient had a raised calcium level and underwent an uneventful laparotomy after initial treatment with furosemide. In comparison, our patient had a sealed perforated peptic ulcer and was managed conservatively. Another case report of a 46-year-old male presenting with abdominal pain secondary to hypercalcemia who underwent successful perforated peptic ulcer repair. This patient had a history of a previous right thyroidectomy, chronic kidney disease stage II, nephrolithiasis, gout, and venous thromboembolism [16]. The exact mechanism by which hyperparathyroidism might be linked to a higher prevalence of peptic ulcer disease in patients is not entirely clear, and no difference in calcium, gastrin, and parathormone serum levels between patients with and without peptic ulcer was observed by Gasparoni et al [20].

Chung et al. reported that about 40%-80% of PPU will spontaneously seal with conservative management known as the "Taylor method," consisting of nasogastric suction, intravenous drip, antibiotics, and repeated clinical assessment only, and the overall morbidity and mortality were comparable [13,21,22]. Our patient was also managed conservatively for a sealed perforated peptic ulcer and showed complete recovery.

## Conclusions

Primary hyperparathyroidism is characterized by the autonomous production of parathyroid hormone resulting in hypercalcemia. Hypercalcemia can affect several organs and processes; however, its association with PUD is still not fully understood. A complication of untreated peptic ulcer disease is PPU, which is associated with high mortality if not treated in a timely manner. Based on the case presented, we recommend screening for primary hyperparathyroidism in patients presenting with abdominal pain and a high calcium level to prevent serious complications such as peptic ulcer perforation and acute abdomen.
